# Copredication in Context: A Predictive Processing Approach

**DOI:** 10.1111/cogs.13138

**Published:** 2022-04-30

**Authors:** Guido Löhr, Christian Michel

**Affiliations:** ^1^ Research Group Philosophy and Ethics Eindhoven University of Technology; ^2^ Department of Philosophy University of Edinburgh

**Keywords:** Copredication, Felicitousness judgment, Linguistic intuition, Predictive processing

## Abstract

We propose a cognitive‐psychological model of linguistic intuitions about copredication statements. In copredication statements, like “The book is heavy and informative,” the nominal denotes two ontologically distinct entities at the same time. This has been considered a problem for standard truth‐conditional semantics. In this paper, we discuss two questions that have so far received less attention: What kinds of word representations and cognitive mechanisms are responsible for judgments about the felicitousness of copredication statements? Relatedly, why can similar copredication statements have different degrees of felicitousness? We first propose a cognitive‐computational model of copredication within the predictive processing framework. We then suggest that certain asymmetries in felicitousness judgments can be modeled in terms of a set of expectations that are influenced by higher‐order priors associated with discourse context and world knowledge.

## 1 Introduction: What is copredication and what is the problem?^1^

The term “copredication” captures the phenomenon that we can use a single nominal to denote two or more distinct kinds of entities in the same statement. To illustrate, consider the following examples:

(1) The manager entered the bankrupt bank.
(2) The heavy book is informative.

In the case of (1), a single noun “bank” is copredicated by “entered” and “bankrupt.” While the predicate “entered” is intuitively taken to apply to the building of the bank, the predicate “bankrupt,” in this context, is meant to apply to the more abstract financial institution whose existence conditions are independent of the concrete building that hosts it. In the case of (2), the physical copy of the book is said to be heavy, while only its content can be informative.

The phenomenon of copredication poses a challenge to standard truth‐conditional semantics (cf. Chomsky, 2000; Collins, 2017; Pietroski, 2018). It is not clear what the denotation of a term like “bank” could be such that it refers to the building of the bank if combined with “entered” and the abstract institution if combined with “bankrupted.” If we restrict the meaning of “bank” such that it refers only to the institution, it ceases to be clear what the truth conditions of (1) could be, considering that we cannot literally walk into an abstract entity. Similarly, it is not easy to see what the reference of “book” in (2) could be, considering that a physical object (the book with pages and cover) cannot literally be informative, and the informational content of the book cannot literally be heavy.

It is currently debated what the phenomenon of copredication tells us about the meaning of words like “bank” and “book” and how these terms relate to truth‐conditional semantics. One option is that nominals like “book” refer to objects that can literally be informative as well as heavy (Liebesman & Magidor, 2019). Another option is that “book” refers to a complex object (e.g., Gotham, 2017) consisting of the informational content of the book as well as a physical object with pages and a cover. A third option is to reject the claim that words have a reference and that linguistics can tell us anything about the nature of books or banks (Chomsky, 2000; Pietroski, 2018).

While there has been significant recent interest in copredication with respect to semantics and ontology, an important question is much less discussed (cf. Ortega Andrés & Vicente, 2019): What are the cognitive psychological mechanisms underlying the processing of copredication statements that give rise to acceptability intuitions? An answer should allow us to address questions like: How can we model or account for certain asymmetries in felicitousness judgments? For instance, why do two copredication statements involving the same pair of senses of the nominal produce different intuitions? Why can the order of the predicates or the discourse context alter our felicitousness judgments in the case of copredication?^2^ Note that by addressing those questions, we do not aim at a philosophical theory of linguistic meaning. Instead, we develop a plausible mechanistic cognitive‐computational model of our felicitousness intuitions with respect to copredication.

The key question we pursue in this paper is as follows: How can we best model our linguistic intuitions based on which philosophers and linguists often draw conclusions about (psychologically significant) word and sentence meanings as well as speakers’ ontological commitments? We will build on work by Ortega Andrés and Vicente (2019), who argue that copredication statements sound felicitous to us because words elicit a body of information or a “coactivation package” that contains the information needed to understand words and sentences. The reason why (2), for example, sounds felicitous is that “book” makes available information about both the abstract content of the book and the physical object that contains the content. We pick up on this idea but supplement it in two important ways.

First, we integrate it into the so‐called “predictive processing” (PP) framework. PP pictures the mind as a “prediction machine.” Contrary to a traditional view of cognition, the PP model construes perception and cognition in general and linguistic understanding specifically (see, e.g., Pickering & Garrod, 2013) and not as merely a passive interpretation of sensorimotor input but as involving active predictions of this input. We argue that information packages can be understood as “expectation hierarchies.” These are complex networks of representations that correspond to expectations at different levels of complexity and abstraction. We call those expectations “priors.” Based on this view, we propose a model of the mechanism underlying linguistic acceptability intuitions.

Second, we go beyond Ortega Andrés and Vicente (2019) by tackling a problem with their information package approach: Their approach has difficulty accommodating asymmetries with respect to acceptance intuitions. Why do some copredication statements sound infelicitous even though the same information is available as in similar felicitous statements? Our model improves the information package approach by introducing different prediction layers and by modeling discourse context. So, while Ortega Andrés and Vicente (2019) assume that information packages are relatively rigid conceptual structures, our model allows for significant context sensitivity, which is not only independently supported by empirical evidence but also accounts better for our linguistic intuitions.

This paper is structured as follows. In Section 2, we discuss the information package approach to copredication in more detail and point to a drawback of the account of Ortega Andrés and Vicente. In Section 3, we introduce the core ideas of the PP framework and propose an account of “information packages” in the form of “expectation hierarchies.” In Section 4, we cover cases of felicitous and infelicitous copredications and discuss the context sensitivity of felicitousness judgments. We also briefly discuss coprediction order effects (Murphy, 2021a, 2021b) and how they can be modeled with our framework.

## 2 The information package approach to copredication

It is largely uncontroversial—and we do not depart from this view—that word forms make available so‐called “bodies of information” or “information packages,” which inform our linguistic intuitions (e.g., Machery, 2009; Vicente, 2021). When the hearer encounters a copredication statement, her cognitive system must combine these information packages in a way that generates intuitions about sentence meanings. We call this *the information package view*. A *philosophical* theory of copredication explains how this body of information relates to linguistic meanings. In contrast, a *psychological* model of copredication specifies the structure and the cognitive content of such information packages and how the system processes them.

Ortega Andrés and Vicente (2019) recently applied the information package view to copredication (see also Vicente, 2021). According to the authors, words make available so‐called “coactivation packages” that contain “senses” or pieces of information, which are closely related by “explanatory, realization relations” (2019, p. 14). For example, the word “book” allows for copredication with the content/physical object alternation in “The heavy book is informative” because both senses are available in the same coactivation package of “book.” The interpretation of copredication then works by pairing each predicate with one of the two coactivated senses (2019, p. 5).

The information package view addresses at least a part of the question of why some combinations are felicitous while other combinations are not. Why can we say that the book is heavy and informative? The answer is that the information immediately available after hearing “book” in a given context contains both relevant senses: the book as content and as a physical object. This is also the reason why we do not consider “heavy” in this context to be used metaphorically, for example, heavy in the sense of sad or intense. Since we know that books have both content and a physical realization, we assume, in the right context, that the speaker means that the *physical* book is heavy, not its *content*.

While we do not disagree with the overall approach of the coactivation package view to copredication, there remain two crucial open questions that we want to address: What determines which senses or information are selected and combined in any given context? Why does the same information package allow for some combinations but render other similar combinations infelicitous? Consider the following statements:

(3a) The newspaper has been attacked by the opposition and was publicly burned by the demonstrators.^3^
(3b)? The newspaper has been attacked by the opposition and fell off the table.
  
(4a) The bank went bankrupt and issued a statement.
(4b)? The bank went bankrupt and flew to the Cayman Islands

According to the coactivation package view, (3a) and (3b) should be equally felicitous. “Newspaper,” in this view, makes available in both statements the same coactivation package consisting of the senses, *newspaper producer* and *newspaper copy*. But why does (3a) sound more felicitous than (3b)?^4^ Similarly, why does (4a) sound better than (4b) if in both cases the relevant coactivation package is the same and, therefore, the senses *bank‐institution* and *bank‐staff* are both readily available?

This problem has recently been observed by Collins (2017, p. 691) in response to theories that take polysemous words to refer to complex objects or dot objects:

*The Times*, let's suppose, has the dot‐object [material institution], but *The Times made most of its revenue from advertising and blew away* is badly zeugmatic. I take it to be an outstanding problem for any account of polysemy in general and copredication in particular why some constructions are acceptable, finding a ready interpretation, whereas others are zeugmatic.

The question of which statements sound acceptable and which statements sound odd is a psychological one—whatever the meaning of words turns out to be. So, we focus here on cognitive processing and subjectively experienced linguistic intuitions and do not discuss the nature of linguistic meaning understood more abstractly (e.g., as meaning as it figures in truth‐conditional semantics). In other words, we take a cognitive psychological stance and do not commit to any philosophical theory of meaning. We argue that the PP framework, which is an increasingly influential framework in cognitive science, can help us model the cognitive processes underlying the processing of copredication statements.

We also worry that Ortega Andrés and Vicente's coactivation package view tends to rely too much on a rather traditional view of linguistic processing. First, linguistic stimuli are processed in a relatively passive way by feature aggregation, for instance, from basic visual features (e.g., a pixel pattern in the retina) to more complex visual shapes to some semantic representation. Second, Ortega Andrés and Vicente's account uses a relatively rigid knowledge structure consisting of discrete pieces of information, namely, senses or concepts, as components that are coactivated by *default* (see Machery, 2009, for this kind of invariantism).^5^ This view arguably leaves little room for context sensitivity. The mainstream in cognitive science, however, is moving toward more flexible, context‐dependent structures (Casasanto & Lupyan, 2015; Kiefer, 2018; Pulvermüller, 2013; see also Löhr, 2017; Michel, 2020). Moreover, expectation or prediction‐based models, as advocated by PP, are becoming increasingly influential in language processing.^6^

Considering recent evidence and theoretical support for context‐dependent bodies of information (e.g., Barsalou, 2009, 2011; Hoenig, Sim, Bochev, Herrnberger, & Kiefer, 2008; Lebois et al., 2015; Ludlow, 2014; Ludlow & Armour‐Garb, 2017; Michel, 2020), we suggest that different senses are not merely simultaneously activated. Rather, we argue that an information package can be understood as an expectation hierarchy defined by its *root node*. Different subparts of that information package are made available depending on the context. Copredicated nominals then do not select different senses, each of which is adequate for one predication. Rather, a certain portion of the information package that is selected can be used for both predications. In other words, we suggest that successful copredication does not involve pairings of coactivated senses with predicates. Instead, it involves the selection of one single and sufficiently abstract representation that is compatible with both predicates.

## 3 PP and information packages

### 3.1 The PP framework

PP is a neurocognitive‐computational framework that construes the mind as entertaining a *hierarchical probabilistic generative* model of the world with which it continuously predicts its sensory input (Clark, 2013, 2016; Friston, 2010; Hohwy, 2013, 2020). In contrast to more traditional views of cognitive architecture, the mind is not viewed as a mere passive analyst of incoming stimuli but as an active prediction machine. This mental model is continuously being improved based on the processing of its prediction errors with the aim of minimizing these prediction errors in the long run and on average.

The PP model is *hierarchical* in the sense that it is composed of various layers of representations. Each layer generates expectations or predictions, which are compared to the signals from the level immediately below. This prediction cascade extends from the highest levels in the neocortex down to the lowest‐level sensorimotor areas. The higher‐level layers make more abstract predictions, that is, predictions with less detail corresponding to more compressed information and represent patterns of larger temporal and spatial scales. In this way, the model tends to replicate the causal structure of the world, which is the source of the sensory signals. Because the brain's model generates proactive hypotheses about the sensory causes, it is also called a *generative* model.

The layers contain *prediction units*, each of which consists, on a neural level, of a pair of a so‐called *representation unit* and an *error unit*. The prediction unit generates a prediction signal that is fed downward and an error signal that is fed upward in the hierarchy of representation layers (see, e.g., Bastos et al., 2012; Kanai, Komura, Shipp, & Friston, 2015; Keller & Mrsic‐Flogel, 2018; Weilnhammer, Stuke, Sterzer, & Schmack, 2018). The error signals provoke updates of the model to reduce future prediction errors.

The system also contains an *error weighting mechanism*, which uses estimates of the precision of the signals to tune the error signals up or down. We do not want the brain to update the model based on unreliable sensory information; therefore, this mechanism can suppress error signals generated by unreliable input. For instance, in a foggy environment, we can rely less on our visual input (we might more easily mistake a cat for a dog). Therefore, we need to give greater consideration to prior experiences and knowledge (i.e., expectations) in our judgments. The error weighting mechanism therefore regulates how we should balance expectations and sensory evidence depending on the situation.

The model is *probabilistic* because it represents states of affairs as probability distributions and carries out approximate Bayesian inference (which is realized by prediction error minimization). Representations at level *N* play the role of predictions or priors for representations at level *N* − 1. Through Bayesian inference, prior beliefs are updated to posterior beliefs to better match the evidence. Then, the posterior beliefs become the new prior beliefs. If the evidence matches the predictions (priors) in all layers, then the prediction error is minimized, and the brain achieves a temporal error minimizing state.

The revisionary element of PP is that what we perceive, grasp, or represent as being the case (linguistically or nonlinguistically) is materialized as predictions. I perceive an apple on the table and grasp (and believe) this fact because my model has predicted an apple on the table—a situation that is compatible with the incoming sensory input and other prior beliefs. We could say that the brain constantly hallucinates, but those hallucinations turn out to match the environment well (under normal circumstances). Similarly, if I hear that you said that you have a new cat because given the context and the stimuli received, this interpretation is most coherent with my network of expectations.

### 3.2 Information packages as expectation hierarchies

We want to apply the PP framework described in the previous section to model copredication sentences. Central to our proposal is how “information packages” associated with a word are structured. Once we have such an account in place, we can show how felicitousness intuitions arise via the violation of the expectations encoded in the information package of the relevant nominals.

**Fig. 1:**
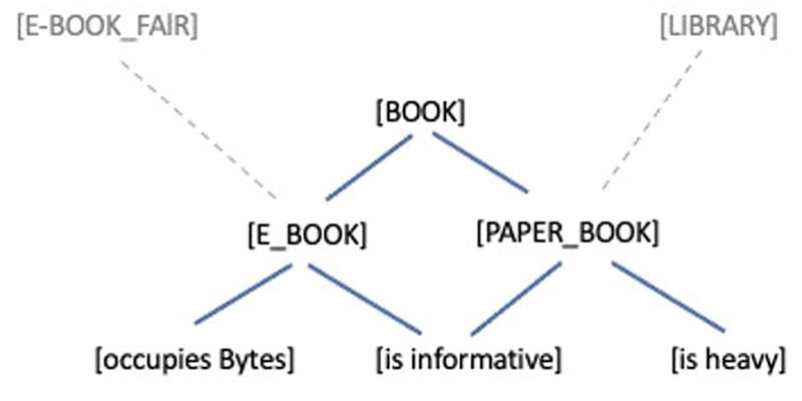
Toy example for the “information package” in the form of an “expectation hierarchy” associated with the word form “book.”

We argue that the PP view provides a novel and empirically plausible model of information packages. An information package associated with a word on the PP picture, as we propose, consists of nodes (in squared brackets) that are connected in a hierarchical tree‐like structure that we call the “expectation network” (see Fig. 1). The expectation network is identified by its root node, that is, the prediction unit “at the top” and from which all other lower‐level (i.e., more specific and concrete) child nodes are connected, forming the hierarchical structure. All these nodes correspond to the abovementioned “prediction units” implemented as neural assemblies. Let us more slowly explain the structure of an expectation network with the example “book.”

Assume that we have a node [BOOK] in the highest (most abstract/compressed) layer of the hierarchy. This node is the root node of the whole information package related to the entity denoted by the word “book.” All (lower level) child nodes emanating from [BOOK] form part of the information package of “book.” For instance, assume [BOOK] is connected to the lower‐level child nodes [PAPER_BOOK] and [E_BOOK]. The node [E_BOOK] is the root node of the more specific information package E_BOOK. The node [is heavy] is the root node of the information package of being heavy, which is connected to the node [PAPER_BOOK], and so forth.

The crucial feature of the information package structure corresponding to the word “book,” that is, the root‐node [BOOK], is that it can relate to the lower‐level nodes [E_BOOK] and [PAPER_BOOK] in terms of different probabilities. In a sufficiently neutral context, both nodes might be similarly probable. Different contexts or higher‐order priors can modify this probability relation. E‐books are expected more at an e‐book fair, while they are probably not expected in a traditional university library (again, this depends on one's previous experience and world knowledge). The higher the probability for a more specific kind of thing or event is, the more “concrete” the expected stimulus becomes.

When hearing “book” in a sufficiently neutral context, our expectations remain rather “abstract.” For instance, again, if the relation between the lower‐level nodes and the root‐node of [BOOK] is rather balanced, then we have a very schematic representation of “book.” In specific contexts, [BOOK] might increase the expectations of specific subpackages such as [PAPER_BOOK] or [E_BOOK]. These subpackages can be made even more specific by including additional nodes further down in the hierarchy. They become more specific once the expectation relation increases for them while decreasing for the other nodes.

In PP terms, once [BOOK] is predicted, we are not surprised to hear or read about e‐books or paper books. At the other extreme, if the path [BOOK]/[E_BOOK]/[occupies Bytes] is predicted, we have a more specific representation for “book” where the features [E_BOOK] and [occupies Bytes] are cognitively salient. The depth of prediction is context dependent. Context is represented in the model as a set of priors as well. When visitors at an e‐book fairly use the word “book,” the node [E_BOOK] receives a higher probability than [PAPER_BOOK]. This is because some situational higher‐level priors (e.g., BEING_AT_AN_E‐BOOK_FAIR) represent our awareness that we are at an e‐book fair and makes available an expectation about e‐books, not physical books. In a library, the librarian's use of the word “book” might increase the probability of the prior [PAPER_BOOK] because the awareness is represented by, for example, BEING_IN_A_LIBRARY, which is a prior that makes us expect paper books. When someone is wondering how much storage is needed, the subpackage [E_BOOK]/[occupies Bytes] receives a higher probability, and so forth. Any given information package, such as [E_BOOK], is embedded in the overall network of priors constituting the brain's model of the world. Priors outside the information package [BOOK] (like BEING_AT_AN_E‐BOOK_FAIR) constitute a context‐sensitive influence on the package.

At this point, a question might arise about how those expectation hierarchies are constructed.^7^ This question reduces to the more general empirical question of how the PP model of any individual is built. What can be said here is that PP has certain empiricist tendencies in the sense that the mental world model is constantly adjusted to the sensory input from the world. One might expect that over time, it will structurally correspond to the world, at least in aspects relevant for the survival of the cognitive agent. The world contains regularities on different spatiotemporal timescales that cause the barrage of signals that hit our sensory periphery. The hierarchical generative model is then a model in the form of interconnected prediction units representing the model variables relevant to predicting the sensory inflow (those variables we call priors or expectations). Despite the empiricist tendency, PP is also compatible with the view that many expectations are innate. After all, it is difficult to see how a model can get off the ground without at least some initial biases.

The core idea that we will develop in the rest of the paper is that copredication statements are felicitous if we can minimize the prediction error in the information package structures involved in the processing of these statements. However, before we can characterize in detail the felicitousness of copredications, we need to provide additional background on how prediction error minimization in language processing works according to the proposed PP model.

### 3.3 The holistic nature of prediction error minimization

It is crucial for our proposal to understand the interplay of top‐down and bottom‐up information flow^8^ in the PP model. There is a constant process of adjusting priors and suppressing error signals, such that the brain reaches a prediction error minimizing state in the *entire hierarchy*. Such a state then corresponds to a certain mental state, like a perception or a belief.

This view can best be illustrated by considering how our brain processes visual input. We must deal not only with incoming stimuli from the retina but also with expectations or priors (e.g., Rao & Ballard, 1999). Both types of signals must be related in such a way that they match. If a reliable external stimulus is unexpected, that is, inconsistent with the priors, the priors are likely to be adapted such that the error signal will be minimized. If the external stimulus is estimated not to be reliable (e.g., in a dusty environment or when it is dark), the priors will be given priority, and the error signal will be suppressed.

Take as an example the visualization of faces. To recognize something as a face, information passes through various stages in the neural abstraction/compression hierarchy of the brain. Imagine looking at a screen that presents you with pictures of different faces. The brain will immediately make predictions as to what kind of stimuli you will be presented with. Here, different layers of the brain represent stimuli at different degrees of abstraction/compression. An initial neural layer in the retina represents a pixel field. In a subsequent layer, neuron assemblies can recognize pixel patterns as elementary edge forms. Higher in the hierarchy, we have representations of more complex lines and shapes. Finally, there is a layer in the visual cortex with neurons sensitive to faces (regardless of, e.g., specific light conditions, head positions, etc.) and that “assumes” that the incoming information is about faces. The face detector is so keen on detecting faces that it can make you see faces where there are none.

One may erroneously see a face when briefly exposed to a vague visual stimulus, for example, a cloud. This can be explained by an expectation effect (e.g., Barik, Daimi, Jones, Bhattacharya, & Saha, 2019; Kok, Brouwer, van Gerven, & de Lange, 2013; Salge, Pollmann, & Reeder, 2020). At some point, however, you realize that what you are seeing is not a face given that you have background beliefs (priors), according to which it is highly unlikely that a cloud literally has a face. The two contradicting predictions (the sensory information that it is a face and the prior expectation that it is not a face) produce an error signal that the brain needs to reduce. Given that one did not look carefully enough, the precision of the perception (“it is a face”) is estimated to be low, the error signal is suppressed, and the prediction (“it is not a face”) now prevails.

The representational structure and mechanism of PP can also be applied to higher cognition such as language and conceptual thought. Word recognition works like face recognition. Once a stimulus, say a certain two‐dimensional shape drawn with a pencil, is recognized not as a face but as a familiar word, it immediately changes the probability of future perceptual stimuli. Clearly, recognizing a shape as the printed word “bird” in a certain context, say when sitting in a psychological experiment staring at a screen, will prime you to generate certain expectations, for example, of seeing other bird‐related words or seeing or hearing bird sounds. The word “bird” serves as a label for the corresponding information package [BIRD] and hence plays the role of a prior that generates expectations related to birds. Translated to the PP model, this means that an adjustment of priors is happening such that when something is seen after exposure to the word “bird” and a bird is seen, the error signal is minimal. When something is seen and it is a horse, the error signal will likely be higher and the prior on a higher level needs to be changed to [HORSE]. This assumes, of course, that the horse is clearly seen, and the visual stimulus is therefore assigned a high‐precision estimate. The expectations related to the word stimulus “bird” are strongly constrained by the mental expectation model, and of course, not *everything* bird‐related is expected. When the screen suddenly disappears in smoke and a huge peacock appears instead, the surprise (and hence the prediction error signal) will be large.

Importantly, for the present paper, predictions are made all the time when processing statements in incremental steps. When having processed a statement partially and having, for instance, recognized the incomplete sentence fragment, “The train is colored...,” the chances are high that a Dutch person will expect the next word to be “yellow.” This is because in The Netherlands, trains are typically yellow, and therefore, the Dutch person has a mental model that contains an information package [TRAIN] with the color feature [YELLOW] as being highly probable. When the statement is continued with “red,” a Dutch person will likely be slightly surprised, that is, a small error signal will be generated because [RED] is not a feature of the information package of [TRAIN], which has a high probability associated. The error can easily be minimized because, of course, a train can be red (e.g., in a different country or in The Netherlands when we deal with a Coca‐Cola promotional train). An even larger error signal should arise when the statement continues with “sour” because while a train can be red even for a Dutch person, it cannot be sour (Hagoort, Hald, Bastiaansen, & Petersson, 2004). In this case, the information package [TRAIN] does not even have a feature representing smell properties because people in general do not have taste experiences with trains. Given that we have here a sort of category mistake (“sour” cannot be applied to “train”), we deal with a prediction error that is difficult to resolve. This example merely serves to illustrate the core idea of processing statements based on expectations, and the mechanism applied to copredication will be explained in more detail later.

We cannot review all of the available evidence for the general PP model of cognition. However, there is increasing support for the idea that predictions and expectations play a critical role in linguistic and nonlinguistic thought. Neuroscientists have suggested that there is a neural marker, the so‐called N400, which has been observed for violations of semantic and world‐knowledge expectations (e.g., Hagoort et al., 2004).^9^ One advantage of the PP model is that there is no principled distinction between semantic and world knowledge violations, which matches those findings and makes it a parsimonious account. More generally, there is mounting evidence that language is underpinned by predictive mechanisms as posited by PP on all levels of the linguistic hierarchy: phonemes, words, sentences, and discourse (see, e.g., Chow, Lau, Wang, & Phillips, 2018, p. 804; Kuperberg & Jaeger 2016).^10^

At this point, we should emphasize two unique and critical features that the PP framework contributes to the information package account proposed. The PP framework supplies a model of constraints that we will need for our copredication account and for how they work computationally, namely, by prediction error minimization in the network of priors. Furthermore, PP is a holistic approach to cognitive processing that naturally provides resources for an account of the *context sensitivity* of the felicitousness of copredications. Context, as we will see, is operative in the form of priors outside an information package.

Before we turn to the PP account for intuitions of copredication statements, we need an additional critical and PP‐specific ingredient. It consists of the assumption that we can read or listen to a statement in two processing modes, namely, a “shallow” and a “deep” one.

### 3.4 Shallow and deep processing of a statement

Remember that one of the central commitments of the PP framework is a countercurrent information flow: top‐down predictions and bottom‐up “evidence.” Furthermore, PP posits a mechanism to regulate the influence of either of those two directions of processing. If incoming information is estimated to be unreliable (or irrelevant), then prior knowledge has more weight in the predictions. If sensory information is precise but does not correspond to the predictions based on prior knowledge, then the system tends to modify/update the higher‐level predictions.

An idea central to PP is that the cognitive system can regulate whether it prefers updating the higher‐level predictions or the lower‐level prediction that serves as “evidence” for the higher‐level prediction. This leads us to posit two distinct modes of processing a statement within our PP framework: *shallow* and *deep processing*. This distinction is inspired by the influential “levels of processing” framework in memory research (e.g., Craik, 2002; Craik & Lockhart, 1972). Depending on the specific task, the semantic information accessed when processing words can be more or less rich or detailed. This idea has also been taken up by Barsalou, Santos, Simmons, and Wilson (2008) (see also Simmons, Hamann, Harenski, Hu, & Barsalou, 2008), who distinguish shallow, that is, merely syntactic processing of language from deeper processing involving richer sensorimotor simulations.

Within the PP framework, we posit a *shallow mode* of processing of a statement in which the overall understanding of the situation expressed by this statement is prioritized. An overall situation is “understood” when we settle on a prediction in the form of a higher level situational prior. In this mode, we might reduce the influence of certain evidence to minimize the overall prediction error.

In the *deep mode*, what is prioritized is the priors representing the lower‐level evidence for the higher‐level hypothesis, here in the form of words and phrases. In this mode, we tend to hold the lower‐level evidence fixed and update the higher‐level prediction to minimize the prediction error. In other words, in the shallow reading mode, we care about the overall gist of the situation described. In the deep reading mode, we care about the detailed understanding of the words, their denotations, how they fit together into phrases, and so forth.

The PP apparatus supplies tools for modeling those two modes on a cognitive‐computational level through an attention mechanism. Attention is often explained in PP by an increase of the error‐signal sensitivity of the relevant domain (see, e.g., Feldman & Friston, 2010; Hohwy, 2012, 2013). If we attend to the individual words and phrases, we increase the error‐signal sensitivity on the level at which words are represented. If we attend to the situational gist, we increase the error‐signal sensitivity on the level where situational patterns are predicted.

This distinction is psychologically plausible and receives further support from Kahneman's findings that the brain “operates as a machine for jumping to conclusions” with the aim of creating a coherent overall story (e.g., 2012, p. 85). In fact, one can compare this to Kahneman's famous distinctions between “System 1” and “System 2” thought processes.^11^ System 1 is unconscious and quick and might correspond to the shallow processing mode. The objective is to quickly “jump to conclusions” about the overall situation. System 2 is conscious and effortful and might correspond to the deep reading mode. In this mode, we pay careful attention to the relevant distinctions that give rise to the impression of polysemy.

Characteristic of shallow reading of a statement is that certain details concerning words (or grammar) are disregarded given that what is prioritized is the gist of the situation. This has various important implications. First, it allows us to perfectly understand statements with wrong words (malapropisms, e.g., Davidson, 1986) or with grammatical errors. Those errors often go unnoticed. For instance, consider “Moses sentences” (see Erickson & Mattson, 1981, or Barton & Sanford, 1993). People tend to answer the question “How many animals of each kind did Moses take on the Ark?” with “two.” They overlook that it should say “Noah.” It seems that when shallowly reading the Moses sentence, “Noah” is represented rather “sloppily” (in more neutral terms: “flexibly” or even better “abstractly”) as “some biblical person.” This more abstract interpretation of “Moses” is enough to grasp the gist of the situation.

The tendency to “jump to conclusions” on a situational level is highly natural and probably essential given that we are embodied minds that need to survive in an uncertain environment and, hence, need to deal with all kinds of situations all the time.^12^ In the deep reading mode, on the other hand, the details of a statement, that is, words and phrases, are prioritized over its overall gist. We read more carefully and conscientiously with awareness, for example, of denotational nuances of words. However, then it can happen that we do not manage to integrate the words into an overall sentence meaning. We might understand each word, but we do not understand the whole sentence.

What the system tries to achieve is an optimal balance between the two modes of processing. If too much focus is placed on the detail, that is, words and their exact “sense” and how they combine with predicates, one might not see the forest for the trees, that is, one might not comprehend the complete sentence. If we are too sloppy with respect to the details, the way we end up interpreting the statement might have little to do with what the statement actually says, which may harm communication. When we read difficult texts, we often alternate between the two modes. We try to understand in detail some complicated sentences and then step back to grasp the overall message or big picture. The same is true on the statement level with regard to words.

We now have the two essential ingredients derived from the PP framework, namely, the expectation hierarchy structure and the two modes of reading in place. These allow us to formulate a model for intuitions about the felicitousness of copredication statements.

## 4 A PP approach to copredication

In this section, we work out the PP‐based approach to copredication. We proceed in three steps. First, we discuss cases of felicitous copredications and provide a characterization of felicitousness (Section 4.1) within the PP framework. Then, we discuss examples of infelicitous copredications (Section 4.2). Finally, we discuss how the PP framework can model the fact that felicitousness intuitions are context‐dependent (Section 4.3). This suggests an answer to the problem of asymmetric felicity intuitions that is unanswered by Ortega Andrés and Vicente's coactivation package account.

Notice that this section presents a *model* of a cognitive architecture underpinning copredication. The plausibility of such a model does not depend on whether the reader finds the reported acceptability intuitions convincing. We focus on examples from the literature, and it may be that some readers have different intuitions. This should be reflected by the way their individual acceptability intuitions are modeled. On an abstract level, felicity judgments are based on world knowledge and innate constraints (or an interplay of the two), which are reflected in our individual cognitive architecture. Note also that intuitions regarding acceptability are not always clear‐cut. We incorporate this idea by referring to different degrees of acceptability (see, e.g., Murphy, 2021a).^13^

### 4.1 Felicitous copredication

Consider the following example:

(5) The school caught fire while it was celebrating Fourth of July.

We argue that copredication statements are felicitous in the PP model if our mind can adjust the network of priors such that prediction errors are suppressed. In the case of (5), for the nominal “school,” we apply a *single* and more abstract prior that can be combined with, say, “caught fire” and “was celebrating.” Therefore, “school” is not interpreted as two different entities but as a single prior that is more abstractly represented and that allows us to expect the two more specific priors. Only after more careful deliberation and conscious analysis (what we call “deep reading”) do we realize that “school” might denote two different entities: One is a school *building* that can catch fire—the other is an *institution* whose anniversary it is and whose members can celebrate.

Fleshed out in more detail, we take there to be an information package with the root‐node [SCHOOL] that is the prior of the two more specific subnodes [SCHOOL_BUILDING] and [SCHOOL_ INSTITUTION]. Let us assume that [CAN_BURN] and [CAN_CELEBRATE] are child nodes of [SCHOOL_ BUILDING] and [SCHOOL_INSTITUTION], respectively. When we read “school” in the shallow processing mode, the error sensitivity (i.e., attention) is increased for the more schematic prior [SCHOOL]. It is decreased for the more specific [SCHOOL_ BUILDING] and [SCHOOL_INSTITUTION]. [SCHOOL] is a prior for its child nodes, which in turn are priors for the nodes representing the predicates. Hence, copredication is successful, as no expectations are violated. [SCHOOL], [CAN_BURN], and [CAN_CELEBRATE] are part of the same expectation hierarchy, and [SCHOOL_BUILDING], [SCHOOL_INSTITUTION], and—by “transitivity”—the predicates [CAN_BURN] and [CAN_CELEBRATE] are expected to a similar degree in this mode (this is because of the reduction of the prediction error sensitivity below [SCHOOL], which creates a degree of “indifference” among the child nodes). By processing in the shallow mode, we quickly get an overall “good enough” (Ferreira & Patson, 2007) understanding of the statement (here: a vivid scene where a fire interrupts the school's celebration). This scene is represented as a strong higher‐level situational prior that now influences word processing.

Now turn to a more careful and detailed word‐by‐word reading or what we call “deep processing.” What changes, compared to the shallow mode of processing, is simply that we focus more carefully on the words (nominals and predicates) and now realize that two different kinds of entities need to be combined with each predicate. In the deep processing mode, the reader focuses her attention on individual words/phrases and each combination of the nominal/predication. This increases the influence of the individual words and de‐emphasizes the overall situational understanding of the statement. In the PP literature, the focus is, again, often cashed out as increasing the error‐signal sensitivity of the relevant domain (see, e.g., Feldman & Friston, 2010; Hohwy, 2012, 2013). When the reader detects the word “school,” the focus is on the word “school” and its combination with the first predicate, that of a fire. In this context, hearing the word “school” strongly increases the error‐signal sensitivity of [SCHOOL_BUILDING] and reduces the expected probability of hearing about schools as an institution. This means that what is expected next is something that has to do with a building.

When we continue reading, we encounter the predicate denoting the celebration. This is unexpected because [SCHOOL_BUILDING] is still the most expected prior without being a prior of [CAN_CELEBRATE]. “School” now needs to be modulated to the prior [SCHOOL_INSTITUION] such that we expect this predicate (minimize prediction error). This process is sometimes (in especially unexpected cases) manifested by a feeling of oddness.

However, even in the deep mode of reading, statement (5) is not entirely incoherent and manifests some degree of felicitousness. This, as we have already suggested above, is because the human mind tends to aim at a situational, that is, statement‐level understanding, and a detailed word‐level understanding is only instrumental. This is plausible in the PP framework. According to some PP theorists, mental predictions serve only one purpose: to secure the survival of the mind‐body system that is thrown into and interacts with an uncertain world (Clark, 2016; Friston, 2010). The dominant level of representation must therefore be the level of situations. Even when we read (5) in the word‐by‐word deep mode, that is, extremely carefully and very reflectively, we cannot escape the automatic force that drives us to interpret the statement on a situation level; we grasp intuitively and immediately that we are dealing with a scene of a celebration that is spoiled by an unfortunate fire.

Let us examine another example of what is considered to be a felicitous copredication to further illustrate the model (Ortega Andrés & Vicente, 2019, p. 16):

(6) Brazil [place] is a large piece of land and Brazil [people] is Portuguese‐speaking and Brazil [government] is a republic and Brazil [economic system] is very high in inequality and Brazil [football team] is always first in the FIFA rankings.

(6) is perfectly well understood, and—if special attention is not drawn to them—the fine distinctions in terms of the “senses” of “Brazil” indicated in brackets go unnoticed. Furthermore, the following copredication statement, which only differs from (6) by mentioning the nominal “Brazil” only once, appears to be felicitous:

(7) Brazil is a large piece of land, Portuguese‐speaking, a republic and is very high in inequality and always first in the FIFA rankings.

According to our model, when hearing (7), the mind engages in shallow reading and is not aware of all of the fine‐grained sense distinctions. In the shallow reading mode, the influence of the lower‐level priors of the prediction is reduced (less attention is given to the details), and the sensitivity of the [BRAZIL] node and higher‐level situational priors is increased (more attention is given to the large situational picture). If the sensitivity of the lower‐level nodes [BRAZIL_PLACE], [BRAZIL_PEOPLE], and so forth, were high, then the mind would struggle to integrate the statement (in PP terms: to predict the overall situation). Only by careful reflection and word‐by‐word analysis of the statement, that is, in the deep reading mode, might “Brazil” be modulated into the more concrete and specific priors [BRAZIL_PLACE], [BRAZIL_SOCCER‐TEAM], and so forth.

We can summarize the case of felicitous copredications with the following condition^14^ (generalizing from two to *n* predications):

*Felicitous copredication condition (FCC)*: There is a prior [*N*] corresponding to the nominal that has *n* child nodes [*N*_1_], [*N*_2_]..., [*N_n_*]. Those *n* child nodes in turn serve as priors for the *n* predicates.

When FCC is fulfilled for a copredication statement, shallow reading can succeed and provides us with a felicitousness intuition. However, note that sometimes shallow reading fails even if FCC is met, namely, in cases where the statement saliently expresses a *spatial or temporal separation* of the entities that the different senses represent (see example 5b in Section 4.2). However, this is exactly what we should expect to happen.

### 4.2 Infelicitous copredication

From FCC, one can derive a condition for infelicitous copredications, namely, simply by its negation: if FCC is *not* fulfilled, then a copredication is infelicitous. As it turns out, copredicative statements might be infelicitous for a range of reasons. However, we want to focus only on the interesting cases where infelicity has to do with the existence of different “senses.” Consider again the following copredication statements that are considered infelicitous in the literature (e.g., Collins, 2017; Vicente, 2021).

(3b)? The newspaper fired the editor and fell off the table.
(5b)? The school caught fire when it was on excursion.

In the case of (3b), infelicitousness depends on the existence of two different senses of “newspaper,” newspaper‐institution, and newspaper‐copy. On our account, what is missing here is a common, more abstract parent prior [NEWSPAPER] for [NEWSPAPER_institution] and [NEWSPAPER_copy] (contrary to [NEWSPAPER_copy] and [NEWSPAPER_content], which do have such a prior). When we think of a newspaper as an institution, the scenario in which its product falls off the table is highly unexpected, and no obvious adjustment of priors is possible to minimize the prediction errors.

Similarly, statement (5b) is infelicitous because once the first part has been grasped, the second part is unexpected. The phrase “caught fire” is expected by the prior [SCHOOL_building], while “was on excursion” is expected by priors such as [SCHOOL_faculty] and [SCHOOL_students]. These three priors are child nodes of [SCHOOL]. Therefore, the statement fulfills FCC. However, a modulation of “school” toward the more abstract [SCHOOL] through shallow reading is blocked here as opposed to example (5). We cannot easily ignore the sense distinctions through shallow reading. It is precisely by grasping the overall situation/scene (which is the whole objective of shallow reading) that we become aware of the two different senses. It is salient in statement (5b) that [SCHOOL_building] and [SCHOOL_students] are *separate* entities, precisely because the statement's content explicitly expresses spatial separation. Therefore, we cannot suppress that “school” refers to different things (building vs. people) by just representing the more abstract [SCHOOL] and using it for both senses at the same time.

We suggest then that a copredication is *infelicitous* if a conflict between the expectations evoked by the statement cannot be resolved, that is, a significant prediction error remains. This persisting prediction error exceeds a threshold such that we become aware of it, leading to the infelicitousness intuition. In *felicitous* copredications, we shallowly process a structure consisting of a more abstract parent prior and two (or more) different child nodes, which in turn are priors for the predicates. The modulation of the attention toward the parent, rather than the child priors, “resolves” the clash of expectations.

Couched in PP terms, infelicity is a consequence of the failure of the brain to settle the network of priors in an error‐minimizing equilibrium. Error signals can be minimized by changing priors in adequate ways. However, priors cannot be modulated arbitrarily because the relations between the priors on different levels are expectations, that is, constraining relations. Certain priors with high‐precision estimates are less “flexible” than others. Therefore, the configuration of priors can turn out to be such that prediction errors above a threshold remain because of inconsistencies between priors. This generates a phenomenology of unexpectedness or oddness. This cognitively unsatisfactory situation will usually lead us to undertake further efforts to suppress conflicts of expectations by adjusting priors at an even higher level. There are different ways to do this. We could explain the error away by hypothesizing that the speaker has not expressed herself correctly or lacks linguistic capabilities. If that were the case, this high‐level situational prior would lead to a suppression of the lower‐level error signals because we cannot rely on the sentence being correct. Alternatively, we might think that we have not understood well and ask for a clarifying statement.

To summarize, in the proposed PP view, a copredication is infelicitous if there is no prior such that the two different priors evoked by the nominal within the context of each predicate are child nodes of that prior. A copredication is hence infelicitous if it fulfills the following simple *Infelicitous Copredication Condition (ICC)* ^15^:

*Infelicitous Copredication Condition (ICC)*: The priors [*N*_1_] and [*N*_2_], denoted by the same word form, lack a common parent prior [*N*].

We must add here that a copredication is also infelicitous, as already said in Section 4.1, when it fulfills FCC but saliently expresses a spatial or temporal separation of the entities represented by the different priors corresponding to the senses.

### 4.3 Context‐dependency of felicitousness

Thus far, the story is relatively similar to the one offered by Ortega Andrés and Vicente. The authors suggest that in felicitous copredication, two senses of the nominal are “activated” simultaneously as a coactivation package and hence made available to be combined with each predicate. We, on the other hand, have suggested that in felicitous copredications, a more abstract prior makes available an information package in the form of an expectation hierarchy. This more abstract prior can be combined with both predicates.

How can we model the finding that some copredications sound odd while others sound better even if in both cases the nominal is associated with the *same coactivation package*? Ortega Andrés and Vicente do not provide a general solution to this difficulty due to asymmetries.^16^ More recently, Vicente (2021) expressed awareness that a more flexible approach than default coactivation of the senses is necessary.

Let us again analyze example (3) from Section 2, which is problematic for the coactivation approach. It consists of a pair of statements that arguably involves a single coactivation package ([NEWSPAPER_institution] and [NEWSPAPER_copy]) but produces different felicitousness intuitions.

(3a) The *newspaper* has been attacked by the opposition and publicly burned by demonstrators.
(3b)? The *newspaper* has been attacked by the opposition and fell off the table.

Both statements arguably involve the same coactivation package as suggested by the felicity of (3a). Why then is (3b) clearly infelicitous? Positing coactivation packages of “senses” associated with the nominal cannot be the whole story.

We suggest that we need to take the background beliefs into account, which sets up a *context* in which the information packages are processed. The background beliefs have an influence on what part of the package is actually “activated.” This is where the PP model of copredication can play out its strength in modeling context sensitivity. The context sensitivity of information packages is naturally available in the PP model. The priors associated with the nominal information package are embedded in the overall network of priors that constitutes the brain's prediction model. We argue that higher‐level priors *outside* the information package can serve as contextual priors modulating the pieces of information to be “activated” (or in PP terms, have their probability increased). Contextual priors are part of the complete expectation network that needs to be brought into a global optimum for the task of sentence comprehension. When I am in a restaurant, a situation prior is represented in the brain that generates (mostly tacit) expectations of hearing sentences like “Today, we have fresh salmon” rather than “Today, we offer 10% off on tire changes.”

The reason why (3a) sounds at least better than (3b) may be that in (3a), we grasp a situational discourse context in the form of a contextual prior that represents a familiar prototypical scene of a specific way of protesting. Often, symbols of the object/person against whom the protest is directed are burned. We have all seen pictures and videos of flags, books, or photographs that are being burned by an upset crowd. This prototype of protest is a high‐level conceptual pattern represented as a high‐level prior in our model. Once we grasp that the statement is about a protest, a prior is activated, let us say [BURN_A_SYMBOL_AS_PROTEST]. This prior generates an expectation that the mentioned newspaper is being burned. Under the influence of the [BURN_A_SYMBOL_AS_PROTEST] prior, we expect a newspaper‐copy to be burned in virtue of it being a symbol of the newspaper‐institution. For (3b), we do not have such a prior. In (3a), the second part of the statement is easily conceptualized as part of the same event, while in (3b), the second part introduces an event that appears to be entirely unexpected.

Consider again example (5). Vicente (2021) has provided a version of it that is of the form we just discussed to illustrate that the coactivation account needs fine‐tuning. It consists of two statements that invoke the same coactivation package but have different degrees of felicitousness:

(5a) The school caught fire when it was celebrating 4th of July.
(5b)? The school caught fire when it was on excursion.

We have argued above why (5a) is felicitous (at least to some degree). Why is (5b) less felicitous according to the PP account of copredication? According to our model, in (5a), we have a strong situational prior (a party interrupted by a fire) that allows us to expect a building and a celebration (viz., in or at the building). However, in (5b), we do not have a single situational prior that makes us expect both predications. On the one hand, we have a fire in a building, while on the other hand, we have a disconnected situation of a group of people (who happen to be people from the school) on some excursion at whatever location. There is no single situational prior that we can think of that makes us expect a school on fire and a group being on an excursion. After “school caught fire,” we can expect people, but people *in* the school, not people somewhere else who happen to be from the school. As we already suggested, the spatiotemporal relation plays plausibly a role here (see also Vicente, 2021, p. 351). In the felicitous case, we have spatiotemporal coincidence, and in the less felicitous case, we have spatial separation. This spatial separation makes it very salient that the two senses represent different entities. In this case, the mind cannot represent both senses with the same more abstract prior.

Finally, for another illustration of how package‐external priors can influence felicitousness judgments, consider so‐called “predicate order effects” in copredication (e.g., Murphy, 2021a, 2021b; Ortega Andrés, 2020; Ortega Andrés & Vicente, 2019). Such order effects involve two statements with the same predications (and hence associated senses of the nominal) but in inverted orders. The statement expressed by one sentence turns out to be more felicitous than the other. There is no space for a full discussion of the whole range of order effects, but we want to show how our account could model an especially interesting kind of order effect involving concrete and abstract senses of the same word.

Consider the following examples discussed by Murphy (2021a, p. 16)^17^:

(8) a. The city has 500,000 inhabitants and outlawed smoking in bars last year.
(8) b.? The city outlawed smoking in bars last year and has 500,000 inhabitants.
  
(9) a. The White House is being repainted and issued a statement concerning taxes.
(9) b.? The White House issued a statement concerning taxes and is being repainted.

Murphy (and Asher in the case of (8)) takes the b‐statements to sound less acceptable than the a‐statements. Again, the problem for the coactivation account is that the same body of information should be available in both cases. As before, we would like to emphasize that we can avoid the question of whether most people agree with the intuitions pointed out by Murphy and Asher. We recognize that not all readers have the same linguistic intuitions and, in particular, experts may have significantly unusual linguistic intuitions given their unique exposure to certain sentences. At least one of us does not hear any difference. Nevertheless, none of this is critical, as we wish to offer a cognitive mechanism that can be used to model both felicitous and infelicitous intuitions even in the case of individual differences. An *explanation* of the differences in intuition that our model captures likely boils down to differences in experience, for example, exposure to this or similar sentences.

Murphy (2021a, 2021b) suggested that we can explain this asymmetry by parsing preferences: We prefer to process first simpler and then more complex semantic structures.^18^ This suggestion is prima facie quite plausible (not least because it follows some common‐sense principle of “incremental effort”). However, various observations suggest that an alternative interpretation is worth considering. First, take the word “school” and one of its abstract (institution) and concrete (building) senses. According to Murphy, the preferred ordering of senses should be school building and then school institution. However, it seems that the notion of school building is not simpler than that of school institution. The former seems to require the more abstract institutional sense of'school'. We are dealing with a *school* building, not any arbitrary building. We suggest that what might be instantiated in cases like (5) is the concept of school building not building simpliciter.^19^ In any case, it is far from obvious whether a linear order can be established at all in all cases; the approach proposed here has the advantage that it does not need this assumption.

Second, it has been suggested that children can quickly grasp more abstract features and generalizations before more specific exemplars are presented (e.g., Keil, 2021; Kemp, Perfors, & Tenenbaum, 2007) and that perception of complex objects/scenes often involves first grasping the overall gist before concrete details (e.g., Barrett & Bar, 2009; see also Fillmore, 1975). This at least suggests that conceptual processing, including the processing of concepts within copredication statements, might not necessarily follow a simple‐complex order preference (see also Rappe, 2019, for a PP‐based account of sentence processing in this sense). Now, Murphy explains order effects not by appealing to abstraction but by semantic *complexity*.^20^ However, abstraction and complexity are plausibly often correlated; abstract concepts seem to be more complex in many cases (e.g., school is more abstract but also more complex—in the here relevant sense—than building). In any case, our aim here is not to argue against Murphy's account but merely to illustrate how the proposed PP model can support alternative hypotheses about interesting copredication phenomena.

The question that interests us is how to model the cognitive underpinnings of linguistic intuitions about copredications. Our model offers a neat implementation of the intuitions about (8) and (9). (8b) simply sounds odd because when reading “The city outlawed” our prediction model “jumps” from the parent node [CITY]—which is connected to child nodes like [CITY_government], [CITY_population], [CITY_geography], and so forth—to the child node [CITY_government]. This is plausible given that it is usually the government of the city that makes the laws; hence, [CITY_government] has a high conditional probability given [CITY] and the context of lawmaking. From the subnode [CITY_government], the predicate “has 500,000 inhabitants” is then less expected. Hence, the statement sounds odd.

**Fig. 2:**
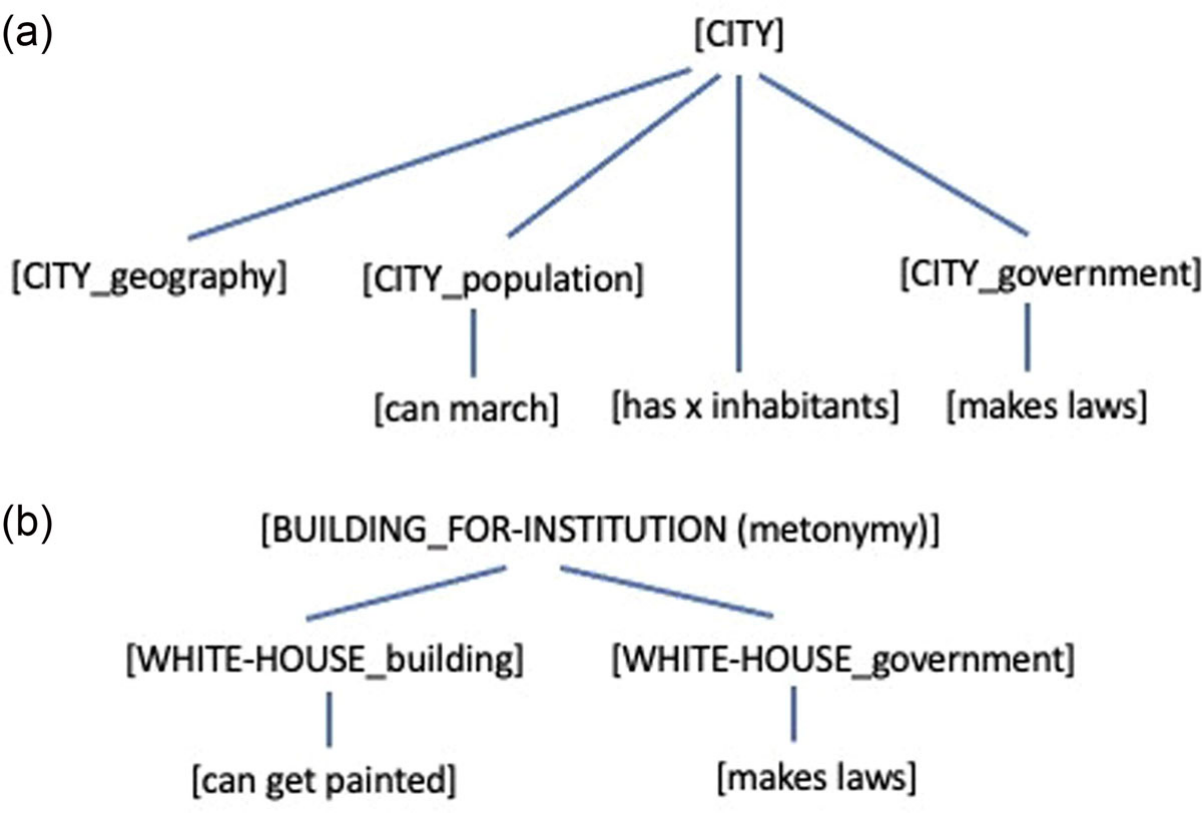
Expectation hierarchies for the “city” (a) and “White House” (b) examples from statements (8a,b) and (9a,b).

This is not the case for (8a) given that now we are talking about the city in its more abstract and inclusive sense ([CITY]) having 500,000 inhabitants. We now expect that more will be said about the city with all its different aspects rather than just one specific aspect of the city as in (8b) (see Fig. 2).

Note that we can expect the predicate “has 500,000 inhabitants” directly from [CITY] but not from [CITY_population]. In fact, “The city marched on the Capitol building and has 500,000 inhabitants” sounds odd. In this sentence, “city” is used in the [CITY_population] sense with “marched”; therefore, having a certain number of inhabitants is expected based not on [CITY_population] but on the more abstract prior [CITY] (see Fig. 2a).

The case in (9) is slightly different, given that “White House” is an intrinsically metonymic expression as opposed to “city.” In statement (9a), “White House” with “is being repainted” evokes the building sense [WHITE‐HOUSE_building]. The second predication “issued a statement” is not expected from [WHITE‐HOUSE_building]; nevertheless (9a) sounds acceptable. This, we suggest, is due to the availability of a building‐for‐institution metonymy that serves as a higher‐level prior. A building‐for‐institution metonymy is nothing more than an expectation relation that allows us to expect an institutional sense given a building sense. In (9b), first the [WHITE‐HOUSE_government] sense is evoked and then the unexpected [WHITE‐HOUSE_building]. It is unexpected because there is (as a matter of linguistic fact) no institution‐for‐building metonymy that can serve as a parent prior (see Fig. 2).

However, these are hypotheses, and much more needs to be said about order effects elsewhere. We also would like to stress again that the explanation of why we have the expectations we have is only a secondary aim of this paper. The primary aim is to provide a model that allows for formulating such hypotheses. In many cases, there might be no "explanation" at all, and the generation of different expectations is simply a consequence of how our cognitive system has evolved to optimally adjust itself to the environment by prediction error minimization. We do think, however, that there is room for interesting generalizations about expectation relations (for instance, metonymy research can be interpreted as contributing to this enterprise). Therefore, there are interesting—but (possibly many) *different*—projects that can provide a taxonomic inventory of all of the possible expectation relations that might do “explanatory” work in a different sense as we have aimed to provide here.^21^

## 5 Conclusion

We have put forward a cognitive‐computational model for felicitousness judgments of copredication statements. The account further develops Ortega Andrés and Vicente's psychological “coactivation package” approach. In their account, the “senses” of the nominal are available in the coactivation packages. In this way, both senses are available for the predications and can be selectively applied. However, this account cannot accommodate cases where the same coactivation package is involved but the felicitousness intuitions are different. What is missing is factoring context sensitivity into an account of the felicitousness of copredications.

We use the framework of PP to provide a model of the structure and context‐sensitive processing of information packages. In PP, cognition is continual prediction making in a hierarchically organized model of the world. The key mechanism for inference and model improvement is prediction error minimization. We have characterized the information packages associated with words as expectation networks consisting of priors (=expectations) at different levels of abstraction. The representations at higher levels serve as predictions or expectations for the representations at lower levels. The information packages are embedded in the huge hierarchical expectation network that constitutes the brain's prediction model.

The core idea is that felicitous copredication is possible because we can make available a single higher‐level prior that is compatible with both predications. This higher‐level prior is a parent prior of the two different priors denoted by the nominal. Infelicitous copredications lack a unifying parent prior. In some cases of apparently infelicitous copredications, higher‐level situational or context priors can contribute to making the copredication felicitous. We have also argued that metonymic constructions can play the role of higher‐level priors, which allows us to explain some of the effects of the order of the predicates on felicitousness judgments.

We would like to close by emphasizing that our focus has been predominantly theoretical and based on explaining linguistic material. More work needs to be done to provide further empirical support for the model presented here, especially the existence of the expectation hierarchies corresponding to priors. Our proposal is empirically testable in principle, as we build on a specific PP model of neural implementation. A possible way to proceed, in principle, would be to localize expectation nodes, correlate them with specific interpretable expectations and intervene on them (e.g., by techniques like Transcraneal Magnetic Stimulation (TMS)) to study the impact on felicitousness judgments. However, this would, of course, require further progress in the study of the way the neurons are connected in the brain and relate to prediction units, and very precise, localized neural monitoring and intervention techniques.

Another approach that could provide further empirical confirmation would be behavioral studies, for instance, cross‐personal and cross‐cultural studies of the variability of felicitousness judgments. As an example that is related to copredications and that involves metonymies, one could try to identify nonuniversal metonymies and then compare felicitousness judgments of copredication statements involving metonymies across different individuals or language communities. Also, cognitive developmental data could be used to test our view. For instance, one could study how the felicitousness judgments of an individual might change after the acquisition of certain metonymic constructions.

## Conflict of Interest

The authors have no conflicts of interest to declare.

## Notes

1 Guido Löhr and Christian Michel contributed equally to the article.

2 Note that we focus on “felicitousness judgments,” that is, one specific, psychological, notion of acceptability. There are others, namely, *grammatical* and *semantic* (in the sense of truth evaluability) acceptability. The relation between the three notions of acceptability is a highly contentious question. Copredication statements seem to be grammatical, and hence are *grammatically acceptabl*e, but it would require further (possibly contentious) assumptions to relate semantic acceptability and felicitousness. This question is highly interesting, but we need not to commit for current purposes to any view of how the semantic and the psychological notions of acceptability are related. Thanks to an anonymous reviewer for recommending to more clearly distinguish those notions.

3 This example is from Nunberg (in Copestake & Briscoe, 1995, p. 55). It is a widely discussed example in the copredication literature. See also Ortega Andrés and Vicente (2019).

4 A referee pointed out that (3a) does not seem to be much better than (3b). As we will make clearer later, we do not deny that there are interpersonal differences in felicitousness judgments, we even expect this to be the case. The example (3a)/(3b) is a standard example discussed in the literature by Vicente and others. We take (3a) to be at least *more* felicitous than (3b), which is sufficient for our purposes as we are interested in explaining why certain statements sound better than others.

5 Note that Vicente and Ortega‐Andrés do not commit to this picture and rather take it as an initial working hypothesis (A. Vicente, personal conversation).

6 By “language processing” we mean processing pertaining to language generally construed: syntax, semantics, and pragmatics.

7 Thanks to a reviewer for suggesting addressing this question.

8 With “top‐down” and “bottom‐up” information processing we mean the following. “Top‐down” refers to the flow of predictions from higher to lower levels of the model hierarchy. In other words, it corresponds to the flow from levels with more abstract and compressed representations farther away from the sensorimotor periphery to layers closer to it. “Bottom‐up” information flow corresponds to the error signals that project from layers closer to the sensorimotor periphery to layers farther away.

9 However, the interpretation of the N400 signal is much more complex than is suggested here (see, e.g., the overview in Kutas & Federmeier, 2011).

10 However, we also acknowledge that PP is far from being a confirmed and mature neurocognitive framework and is still very much in the making. See, for example, Walsh, McGovern, Clark, and O'Connell (2020) for a review of the neurophysiological evidence for PP. The authors conclude that “although the debate about PP's empirical grounding is currently unsettled, the theory can nevertheless be regarded as a milestone in cognitive neuroscience, spurring efforts to recognize the importance of backward connections in the architecture of the neocortex and the role of prediction in sensory processing” (p. 262).

11 Thanks to Beate Krickel for bringing this to our attention.

12 See also Fillmore (1975) for the idea that we grasp a scene description holistically, before we grasp all the details.

13 Murphy provides a wealth of empirical data about the acceptability ratings of many variations of copredication statements. Note that Murphy focuses on aggregate statistics. We are here interested in a mechanistic cognitive account that can also accommodate differences of intuitions across individuals.

14 This condition is an informal summary and is not meant to be a “formalization” of our account in terms of the precise necessary and sufficient conditions.

15 Note that our account is silent concerning what exactly determines whether the required parent prior is available or not. Various authors have discussed patterns that favor felicitousness, like metaphysical relations between the involved senses (see Vicente, 2021). We are skeptical about the possibility of positing a comprehensive system of rules or generalizations that capture those patterns because of the context‐sensitivity of felicitousness. This does not rule out that some generalizations can be identified that are descriptively useful.

16 However, for some specific cases of asymmetries, for example, pairs of sentences involving a word that has a content and a container sense (like “glass” or “beer”) and that appear in a different order, the authors point to *metaphysical dependency relations* as the reason of the difference in felicitousness (e.g., Ortega Andrés, 2020; Ortega Andrés & Vicente, 2019, especially chaps. 6.3 and 6.4).

17 Example (8) is taken from Asher (2011, p. 63).

18 Murphy's experimental data (2021a) indeed shows a simple‐complex order bias for the cases that he has investigated. The question is whether the variable “complexity” plays a central role in a mechanistic cognitive‐computational account of copredication. In other words, Murphy's interpretation might be descriptively fully adequate for the experimental results, but it is still an open empirical question whether complexity is a causal‐explanatory variable. For instance, an interesting question is *why* the parser should prefer a simple‐complex ordering.

19 This discussion benefited from a conversation with Agustin Vicente.

20 Semantic complexity is defined by Murphy via the number of cognitive modules involved in the conceptual processing. A module is here understood as a specific domain of reasoning, like number sense, folk psychology, social reasoning, geometric reasoning, and so forth (see 2021a, pp. 54–56).

21 Other examples of relevant types of expectation relations are coherence relations (e.g., Murphy, 2021a, 2021b) and metaphysical realization relations (e.g., Ortega Andrés & Vicente 2019; Vicente, 2021).
